# Differential ABC transporter gene expression in adult *Dirofilaria immitis* males and females following *in vitro* treatment with ivermectin, doxycycline or a combination of both

**DOI:** 10.1186/s13071-019-3645-y

**Published:** 2019-08-13

**Authors:** Chiara Lucchetti, Marco Genchi, Luigi Venco, Alessandro Menozzi, Paolo Serventi, Simone Bertini, Chiara Bazzocchi, Laura Helen Kramer, Alice Vismarra

**Affiliations:** 1 Dept. of Veterinary Sciences, University of Parma, Parasitology Unit, 43126 Parma, Italy; 2 Clinica Veterinaria Lago Maggiore, 28041 Arona, Italy; 3 Dept. of Veterinary Sciences, University of Parma, Pharmacology Unit, 43126 Parma, Italy; 4 Dept. of Veterinary Science, University of Milan, 20133 Milan, Italy

**Keywords:** *Dirofilaria immitis*, PgP, Gene expression, Doxycycline, Ivermectin, Combined treatment, *Wolbachia*, “Slow kill”

## Abstract

**Background:**

Combination doxycycline/macrocyclic lactone (ML) protocols have been shown to provide a more rapid adulticidal and microfilaricidal effect than either MLs or doxycycline alone, although female worms were reported to have a higher tolerance to treatments compared to male worms. The present study aimed to evaluate how ABC transporters may be involved in the synergic effect of the combination treatment. Adult worms of *D. immitis* were treated *in vitro* for 24 hours with doxycycline (DOXY), ivermectin (IVM) and a combination of both, and changes in the modulation of ABC transporter genes were measured. Levels of doxycycline inside different treatment media, post-treatment, were determined through HPLC analysis.

**Results:**

Quantitative RT-PCR analysis showed the presence of changes in the modulation of ABC transporter genes evaluated in this study. In particular, in female worms, the combination treatment induced a substantial increase in gene expressions, especially of Dim*-pgp-10* and Dim*-haf-4*; whereas in male worms, the greatest increase in gene expression was observed for Dim*-pgp-10* and Dim*-pgp-11* when treated with DMSO + IVM and DMSO + DOXY/IVM. HPLC analysis of the DOXY concentrations in the media after *in vitro* treatments of male worms showed a slight difference between the DMSO + DOXY samples and the combination (DMSO + DOXY + IVM), while no difference was observed among females.

**Conclusions:**

Further studies are required to explain whether the modulation of cellular efflux plays a role, even partially, in the adulticide effect of doxycycline/macrocyclic lactone combinations in heartworm-infected dogs. To the authors’ knowledge, this is the first study to evaluate P-gp expression in adult *D. immitis.*

## Background

*Dirofilaria immitis* is a vector-borne parasite transmitted by different species of mosquitoes and the etiologic agent of canine heartworm disease. The disease is endemic in many parts of the world and is currently spreading to previously unaffected areas [1–6].

Melarsomine dihydrochloride (Immiticide®, Merial) is the only approved adulticidal drug for treatment of patent *D. immitis* infections. Protocols combining doxycycline, which targets the bacterial endosymbiont *Wolbachia*, together with a macrocyclic lactone, are currently recommended either as pre-adulticide preparation (resulting in less severe post-melarsomine complications) [7, 8] or as an alternative to melarsomine when this is unavailable or contraindicated [6, 9].

Doxycycline has various detrimental effects on developing and adult heartworms, including degeneration of oocytes and early embryonic stages, but its adulticide efficacy is low and slow [10]. Macrocyclic lactones (MLs) are highly efficacious against L3 and young fourth-stage larvae (L4) and prevention of disease is based on this activity [11]. Long-term administration of preventive doses of MLs have also been reported as having a so-called “slow kill” effect against adult *D. immitis* (for review see [12]). As with doxycycline, however, the adulticide effect is slow (typically 24–36 months) [13]. Furthermore, the use of macrocyclic lactones as adulticides has been implicated in the development of drug resistance and is no longer recommended [11, 14].

The doxycycline/ML combination protocols that have been evaluated in experimentally and naturally-infected dogs have been shown to be highly effective in clearing infection in a relatively short time [7, 13, 15]. It is not currently known, however, why doxycycline/ML combinations provide a more marked and rapid adulticidal effect than the two drugs alone. Furthermore, in a few studies which evaluated worm populations at necropsy following different combination protocols, female worms were often found to survive drug treatment, suggesting a certain tolerance towards the adulticide effects of this treatment protocol [7, 15, 16].

It is well known that ATP-transporters (ABCTs), including P-glycoproteins, are involved in the active carriage of drugs and other molecules and metabolites across cell membranes [17]. Their role in inducing drug resistance has also been widely demonstrated [18–21]. Recently, Mani et al. [22] reported that macrocyclic lactones are able to modulate P-gp activity in *D. immitis*, with ivermectin being a potent inhibitor. Doxycycline has been shown to inhibit P-gp activity in mammals [23], but there is no available data regarding such interaction with *D. immitis* or other nematodes. The adulticide effect of the doxycycline/ML combinations may therefore be due to interaction with cellular efflux mechanisms. Indeed, if these drugs inhibit P-gp activity, thus allowing for accumulation of higher concentrations within the nematode compared to treatment with either drug alone, eventual lethal effects may be enhanced.

To further explore the effect on cellular efflux, in the present study we determined the expression level of genes coding for P-gp transporters Dim-pgp-10, Dim-pgp-11 and Cel-haf-4 in adults of *D. immitis* treated *in vitro* with doxycycline, ivermectin and a combination of both. We also evaluated doxycycline concentrations in the culture medium following treatment.

## Methods

### Reagents

All reagents were purchased from Sigma-Aldrich (St. Louis, MO, USA), except where indicated.

### Collection and maintenance of *D. immitis* adults

Seven privately-owned dogs that had never received macrocyclic lactones or doxycycline were diagnosed with patent *D. immitis* infection. Following owners’ consent, dogs underwent minimally-invasive surgical heartworm removal [9]. A total of 15 female and 15 male adult worms were collected during surgeries. Extracted worms were washed in HBSS medium at room temperature, checked for viability, counted, sexed, and then placed individually into 50 ml of RPMI medium and stored overnight at 37 °C, 5% CO_2_.

### *In vitro* treatment of adults

Adult parasites were placed individually in tubes with 50 ml of NI media [24]. They were then treated as follows: 1% DMSO alone (DMSO), ivermectin (3.54 nM) with 1% DMSO (DMSO + ivermectin), 1% DMSO in combination with doxycycline (56.5 µM) (DMSO + doxycycline), and a combination of doxycycline (56.5 µM) and ivermectin (3.54 nM) together with 1% DMSO (DMSO + doxycycline + ivermectin). Untreated samples, plain NI media, served as controls. Concentrations of the two drugs were chosen according to their highest plasma concentrations measured after *in vivo* treatments of dogs [25, 26]. Given that the present *in vitro* study attempted to mirror what occurs *in vivo*, drug concentrations were not calibrated based on the parasite’s gender and study media volumes were standardized to 50 ml for both sexes. Each treatment was performed in triplicates, for both sexes. Tubes were maintained for 24 h at 37 °C, 5% CO_2_. Exposure time was chosen based on a previous study on IVM treatments both *in vivo* as well as *in vitro* of other nematodes [27, 28]. Samples were checked for vitality and, after being washed in HBSS, they were promptly frozen at − 80 °C. After the treatment, all media were stored at − 20 °C.

### HPLC for doxycycline medium levels

The concentrations of doxycycline in different treatment media (DMSO + doxycycline and DMSO + doxycycline + ivermectin) post treatment were measured by means of HPLC method as described by Menozzi et al. [29]. A standard curve was prepared with doxycycline in NI media. The internal control selected for the study was DMSO + doxycycline.

For this analysis a Prostar LC Workstation (Varian Co., Walnut Creek, CA, USA), with a Prostar325 UV-Vis detector and a 10 µl loop were used. While for the chromatographic separation a Syncronis C18 analytical column (Thermo, Milan, Italy) (5 µm particle size, 150 × 4.6 mm) was selected. Acetonitrile and 0.01 mol/l trifluoroacetic acid (30:70, v/v) were used as mobile phase, with a flow rate of 1.0 ml/min and an analytical wavelength of 350 nm. The software Star Chromatography Workstation System control v.0.41 by Varian was used.

### RNA extraction

Each frozen worm was homogenized in TRIzol® reagent (Ambion®, Foster City, CA, USA) individually with the help of an electric pellet pestle (Sigma-Aldrich®, Missouri, USA). After homogenization, due to their different starting size (~ 200 mg for females and ~ 100 mg for male), in order to perform the extraction under the best suitable conditions female samples were split into four 1.5 ml tubes and male samples were split into two 1.5 ml tubes. Each tube was used for RNA extraction according to the manufacturer’s instructions of the TRIzol® reagent. The starting volume per each tube was set to 1 ml of TRIzol®. RNA samples were rehydrated into 100 µl of DEPC H_2_O. Concentrations and quality of RNA samples were measured through spectrophotometer analysis (BioSpectrometer® fluorescence with μCuvetta® G 1.0, Eppendorf, Hamburg, Germany). Genomic DNA traces were removed with TURBO™ DNase treatment (Thermo Fisher Scientific, Waltham, MA, USA). A second RNA extraction step was performed, as described earlier, except for the volume of DEPC H_2_O used for rehydration (35 µl), followed by a second analysis at the spectrophotometer. Three hundred nanograms of each final RNA sample was used to produce cDNA by an initial step of AccuRT genomic DNA removal (Abm, Richmond, Canada), followed by second step of OneScript® cDNA Synthesis Kit (Abm). Resultant cDNAs were used as a template for molecular analysis.

### Quantitative reverse-transcription PCR (qRT-PCR)

The sequences of two ABC-B transporters genes (Dim*-pgp-10*, Dim*-pgp-11*) and of one ABC-B half transporter gene (Dim*-haf-4*) have been previously described [30]. Primers sets were tested in order to optimize the efficiency and the dynamic range of the reaction. As endogenous control, the *18S* ribosomal subunit was selected [7]. All primers used for this study are listed in Table 1. Three biological replicates of both sexes were prepared per each treatment of interest. Three technical replicates were analyzed per each biological replicate. The gene expression study was carried out by means of a relative qRT-PCR, where results were normalized, first to the internal control gene (*18S* rDNA) and then to the treatment control (NI alone). The BrightGreen 2× qPCR Mastermix (Abm) was used according to the manufacturer’s instructions. The amplification protocol was as follows: denaturation at 95 °C for 1 min 30 s, followed by 40 repeated cycles (95 °C for 15 s; 59.4 °C for 1 min for Dim*-pgp-10* and Dim*-pgp-11* genes, while 57.7 °C for 1 min for the Dim-*haf-4* gene; 72 °C for 20 s). The final concentration for each primer in all reactions was 0.35 µM. Fluorescence signals were collected at every cycle and to avoid the presence of unspecific products, a melting curve analysis was performed.

**Table 1:** Primers used in the present study to evaluate the expression of the two ABC-B transporter genes and the ABC-B half transporter gene

Gene	Primer name	Primer sequence (5′–3′)
Dim*-pgp-*10	DimmScaf48-cDNA-F8	F: GCCATCGTAGGTCCATCAGGTTCTGGT
	DimmScaf48-cDNA-R12	R: TGTTCAACTGAAACGACCACACGTC
Dim*-pgp-*11	DimmScaf04-cDNA-F6	F: TTAACAGTGTTGATGAAGGATCAAATCC
	DimmScaf04-cDNA-R6	R: ATATTTCGCTGCGGTCTTGTTGG
Dim*-haf-*4	DimmScaf101-cDNA-F5	F: GTGCAAACTCGAGGTTTTGCTGT
	DimmScaf101-cDNA-R2	R: TCCACCTCGAAGACCTCCAGCA
*18S* rDNA	18SQ-F	F: GGGACAAGCGGTGTTTAGC
	18SQ-R	R: GCACGCTGATTCCTCCAGT

*Abbreviations:* F, forward; R, reverse

### Data analysis

Results were presented as the relative change in gene expression (2^−ΔΔCt^) ± standard deviation (SD) of the three biological replicates. All Ct values were managed by CFX Manager software (Bio-Rad, Hercules, CA, USA) and 2^−ΔΔCt^ was calculated according to the Livak method [31]. Results were normalized, first to the internal control gene (*18S* rDNA) and then to the treatment control (NI alone). Three biological samples were analyzed per sex. The standard deviation between each technical replicate was calculated. All standard deviations were lower than 0.3, which is the threshold value for a Ct standard deviation to be considered accurate, as described in the “Guide to performing relative quantitation of gene expression using real-time quantitative PCR” published by Thermo Fisher Scientific [32]. The standard deviation per each 2^−ΔΔCt^ was calculated according to the same manual. Data normality and distribution were tested respectively with Pearson test and two-way ANOVA using GraphPad Prism v.8.0.1 (GraphPad Software, San Diego, CA, USA; http://www.graphpad.com) and *P*-values < 0.05 were considered significant.

## Results

### Determination of gene expression profile after treatment

Quantitative RT-PCR analysis of Dim*-pgp-10*, Dim*-pgp-11* and Dim*-haf-4* gene expression showed very little variation between NI controls (whose values were set to 1) and medium with DMSO alone (whose values ranged between 0.9-fold and 1.4-fold). Thus, results are reported for the other treatments compared to DMSO as solvent control.

**Fig. 1:**
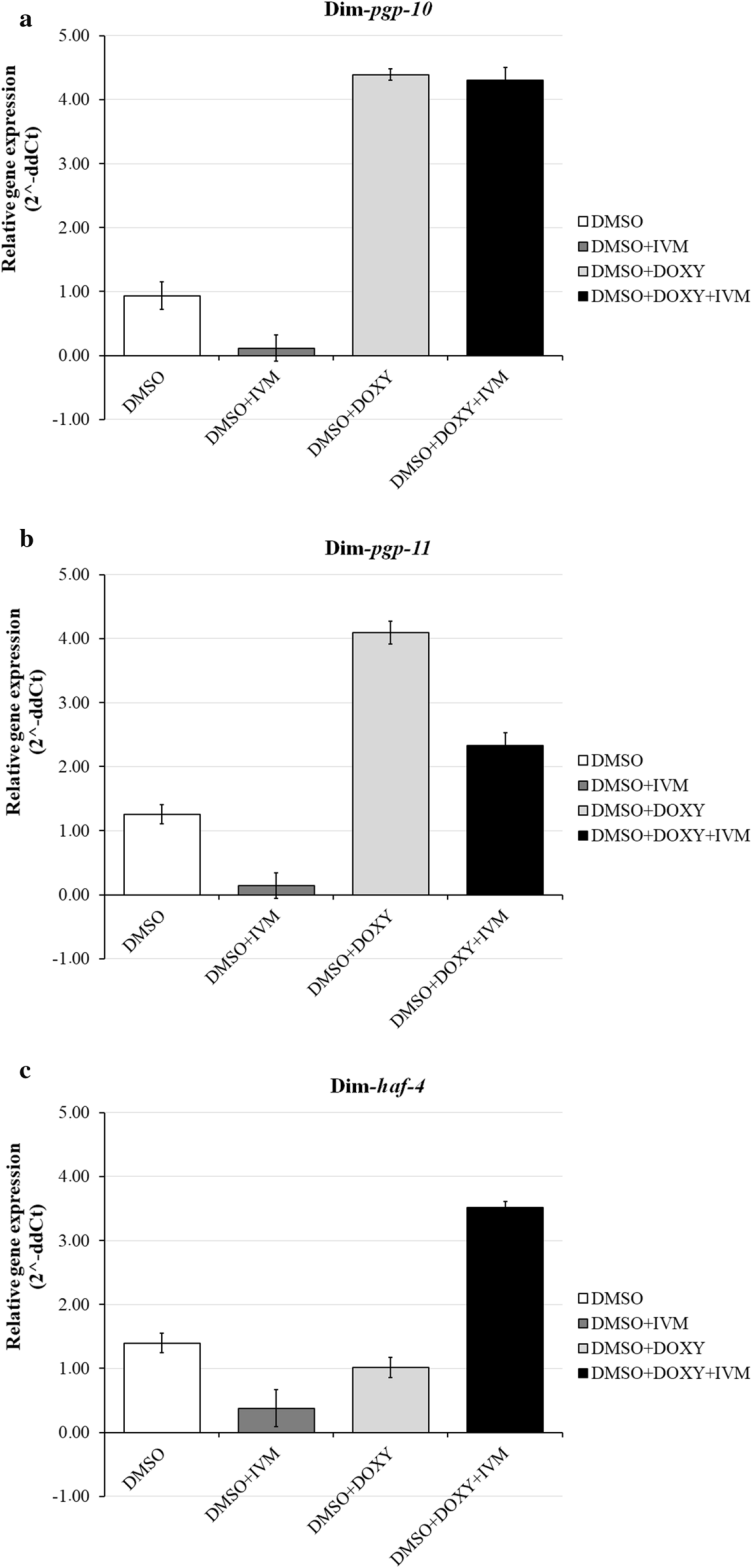
Expression of Dim*-pgp-10* (**a**), Dim*-pgp-11* (**b**) and Dim*-haf-4* (**c**) genes in adult females of *D. immitis* treated with the following: 1% DMSO alone (DMSO), 1% DMSO in combination with 3.54 nM IVM (DMSO+IVM), 1% DMSO in combination with 56.5 µM DOXY (DMSO+DOXY), 1% DMSO in combination with 3.54 nM IVM and 56.5 µM DOXY (DMSO+IVM+DOXY). Results are expressed as fold change of the gene of interest (2^−ΔΔCt^), normalized first to the internal control (housekeeping gene: *18S*) and then to the control treatment (plain NI media). Data are presented as the mean ± SD of the three technical replicates per each biological one

**Fig. 2:**
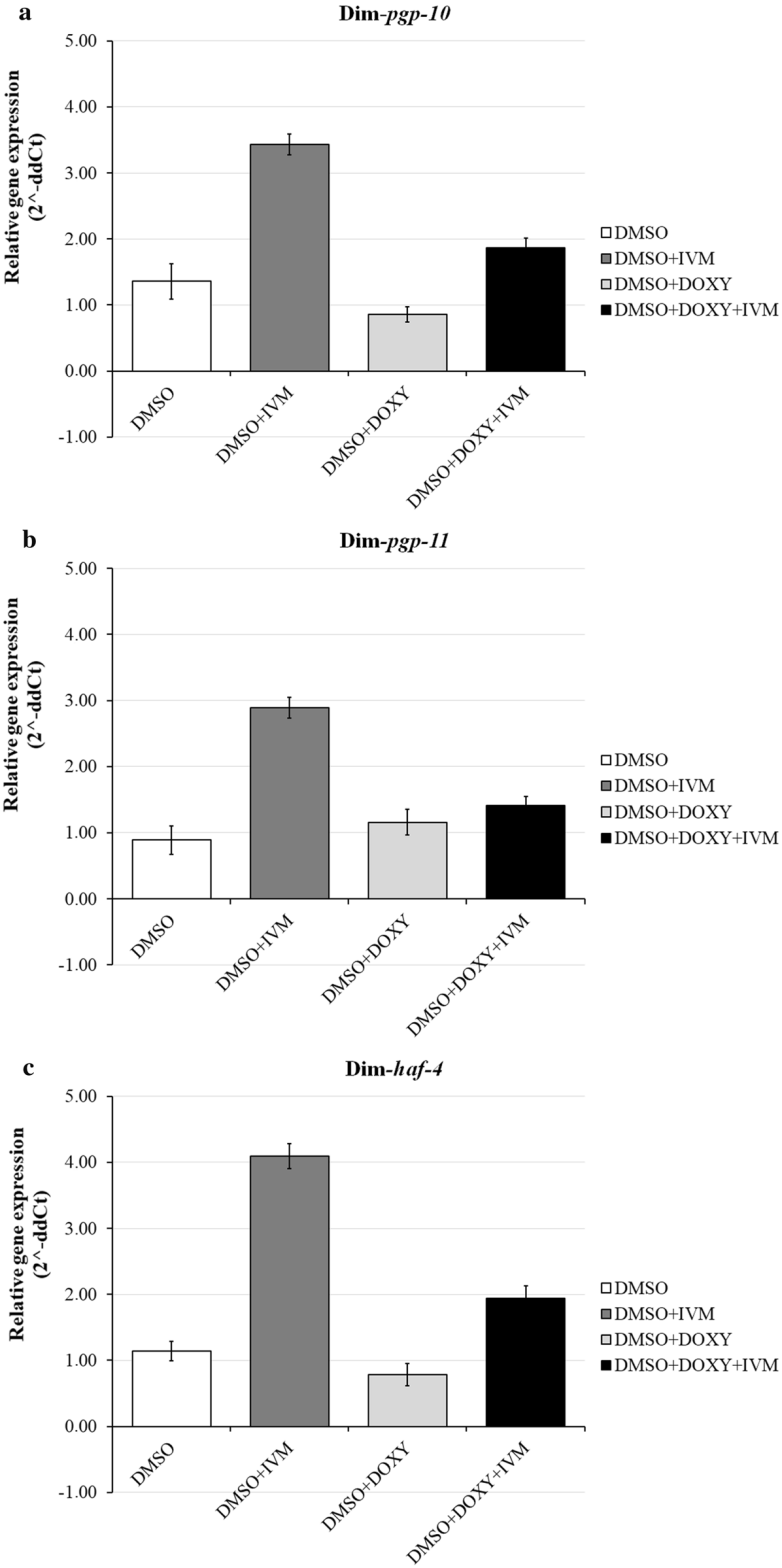
Expression of Dim*-pgp-10* (**a**), Dim*-pgp-11* (**b**) and Dim*-haf-4* (**c**) genes in adult males of *D. immitis* treated with the following: 1% DMSO alone (DMSO), 1% DMSO in combination with 3.54 nM IVM (DMSO + IVM), 1% DMSO in combination with 56.5 µM DOXY (DMSO + DOXY), 1% DMSO in combination with 3.54 nM IVM and 56.5 µM DOXY (DMSO + DOXY + IVM). Results are expressed as fold change of the gene of interest (2^−ΔΔCt^), normalized first to the internal control (housekeeping gene: *18S*) and then to the control treatment (plain NI media). Data are presented as the mean ± SD of the three technical replicates per each biological one

Interestingly, different expression patterns were observed for female *vs* male worms exposed to the different drug protocols. In female worms (Fig. 1), P-gp gene expression was downregulated following treatment with ivermectin (for all three genes, less than 1/4 of the amount of target RNA as the control/calibrator), and upregulated when treated with doxycycline, reaching a fold difference of approximately 4 for both Dim*-pgp-10* and Dim*-pgp-11*. Combination treatment (doxycycline+ivermectin) caused a marked increase in gene expression in female worms, in particular for Dim*-pgp-10* and Dim*-haf-4* (4.3-fold and 3.5-fold, respectively). Expression of the ABC transporters in male worms showed a different pattern (Fig. 2). Exposure to ivermectin caused increase of gene expression (up to 4.1-fold in Dim*-haf-4*), while treatment with doxycycline showed almost no effect on any of the three genes analyzed, whose fold gene expression ranged between 1.16–0.79 (Dim*-pgp-11* and Dim*-haf-4*, respectively). When the two drugs were administered in combination, however, gene expression was upregulated, but to a lesser degree than that observed in female worms (maximum of 1.9-fold observed for Dim*-haf-4*).

None of the differences in gene expression following the various treatment protocols were statistically significant when compared to the negative controls (NI alone). The lowest *P-*value (0.053) was observed between the control and the combination treatment for Dim*-haf-4* gene expression in females. As reported by Amrhein et al. [33], statistically non-significant results cannot be taken as proof of the null-hypothesis. Therefore, we can still consider the results collected in this study as reliable despite their statistical “non-significance”.

### Determination of doxycycline concentrations in treatment media

**Fig. 3:**
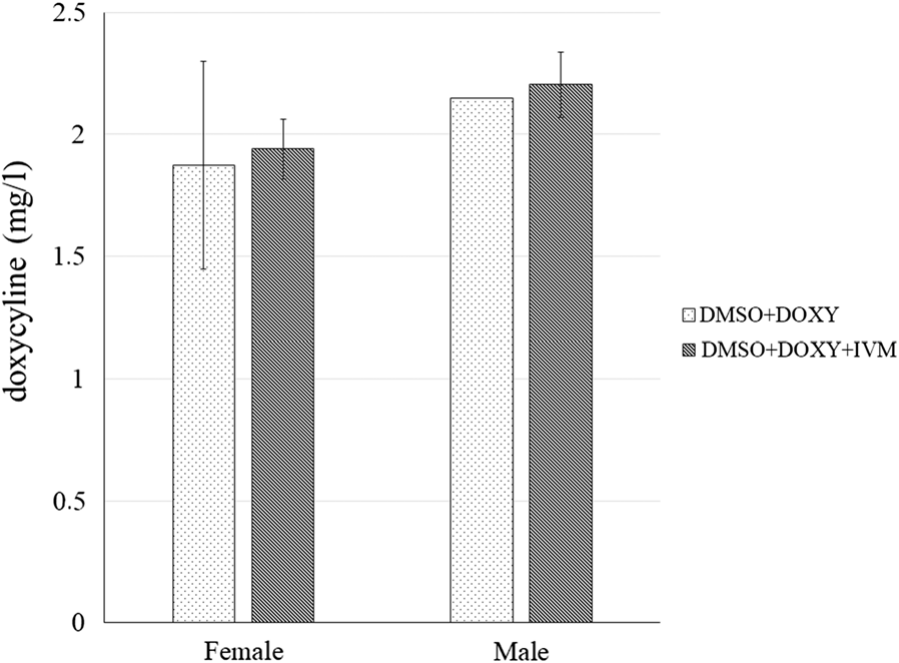
HPLC analysis of doxycycline levels found in different treatment media after adult *D. immitis* treatment. Media analyzed were as follows: 1% DMSO in combination with 56.5 µM DOXY (DMSO + DOXY), 1% DMSO in combination with 3.54 nM IVM and 56.5 µM DOXY (DMSO ± DOXY ± IVM). This histogram shows the average doxycycline concentrations, expressed in mg/l and standard errors calculated from all data collected for three biological replicates

HPLC analysis of the doxycycline concentrations in the media after *in vitro* treatments of worms with either doxycycline alone or ivermectin + doxycycline showed a slight difference between the two sexes (Fig. 3). Media from female worms contained a lower concentration of doxycycline in the media compared to male media for both treatment protocols. When comparing the two treatment media concentrations, the doxycycline samples had lower concentrations of doxycycline than media from all worms treated with the combination doxycycline/ivermectin. None of the observed differences were statistically significant (*P* > 0.9).

## Discussion

Several studies in experimentally- and naturally-infected dogs have reported a marked adulticidal effect of combination treatment with doxycycline + macrocyclic lactones against *D. immitis*. Bazzocchi et al. [7] reported that this adulticidal effect was significantly higher than when either drug is used alone, suggesting a possible synergism between the two. In an attempt to shed light on the possible mechanisms responsible for this, we evaluated cellular efflux gene expression and doxycycline concentrations in the media, following *in vitro* treatment of adult worms.

The most interesting result of the present study is the differential gene expression between male and female worms following treatment. In female worms treated with ivermectin, all three genes were downregulated, suggesting an inhibitory activity of ivermectin on cellular efflux. In male worms, however, there was a 3.4-fold, 2.9-fold and 4.1-fold increase in Dim-*pgp-10*, Dim-*pgp-11* and Dim*-haf-4* expression, respectively, suggesting a stimulatory effect. Mani et al. [22] reported that both ivermectin and selamectin markedly inhibited cellular transport by Dim-*pgp-11* expressed in mammalian cells. Upregulation of P-gp expression, on the other hand, has been reported as a possible mechanism of drug resistance against macrocyclic lactones in several nematodes, including *H. contortus*, *C. elegans* and equine cyathostomins [34–36]. In the present study, worms were isolated from naturally-infected dogs that had not received preventive treatment with either doxycycline or macrocyclic lactones. Furthermore, infected dogs were from a geographical area where the first case of autochthonous infection was reported in 2007 and where regular heartworm prevention is still not practiced routinely [37, 38], making the presence of drug resistant isolates highly unlikely. Therefore, the authors suggest that there is a sex-related modulation in P-gp expression in *D. immitis* following exposure to ivermectin. Interestingly, similar results were reported in 2013 where P-gp gene expression tended to be increased by ivermectin treatment in male *Cooperia oncophora* [39].

This differential expression of P-gp genes is also evident following exposure to doxycycline, causing upregulation in females and downregulation in males. This is particularly interesting given that one of the principal differences between male and female *D. immitis* is the presence in females of a large number of *Wolbachia*, especially in the female reproductive system. It is reasonable to hypothesize that female worms would respond to antibiotic treatment with defense mechanisms aimed at reducing drug concentrations in order to protect the endosymbiont and, by doing so, protecting itself. Indeed, it has been shown that there exists an obligate dependency of these worms on *Wolbachia* for normal embryogenesis and viability [40, 41]. According to a gene expression study of *Wolbachia* from *Onchocerca ochengi*, ATP provisioning was identified as one of the primary contributions of the symbiont to the mutualistic relationship in filarial worms [42]. ABC transporters, including P-gps, are ATP-dependent, and results from the present study would suggest that *Wolbachia* is contributing to cellular detoxification in *D. immitis* females exposed to doxycycline. Why this does not happen in male worms may be due to the location of P-gps. In a recent study, Godoy et al. [43] reported that HcoP-gp9 localized to the uterus of adult females. *Wolbachia* is present in the entire female reproductive apparatus, while it is only present in the hypodermal chords of males. There is, however, no information on the tissue distribution of *D. immitis* P-gps.

The results of the present study regarding gene expression following combination treatment with ivermectin and doxycycline were similar in both males and females, with a more marked upregulation of all genes in females. As mentioned above, those studies which evaluated worm count at the end of combination protocols in infected dogs consistently report at least one or two female worms as surviving, while the death of male worms is 100% [7, 15]. A more intense cellular detoxification in females may explain this phenomenon. Results, however, do not explain why the two drugs together have a higher adulticide effect than the two drugs used separately. This is likely due to the short exposure time (24 hours) to the drugs and/or the low drug concentrations used. Indeed, if these drugs activate P-gps in a dose-dependent, saturable way, it is possible that expression will be modulated differently with longer exposure times and/or higher drug concentrations. The results from doxycycline concentration measurements in the culture media, while confirming a higher efflux/lower uptake of doxycycline in worms treated with the combination protocol, seem to contrast with the results of gene expression in females *vs* males, indicating a possible lack of correlation between gene expression and functionality. A potential summation effect induced by a higher uptake of doxycycline when administered in combination with ivermectin was evaluated by Menozzi et al [29]. They found no statistically different concentration levels of doxycycline in plasma of dogs treated with doxycycline/ivermectin compared to those treated with doxycycline alone. Another study evaluated whether the administration of doxycycline/ivermectin could be responsible for reversing the phenomenon of immunological tolerance towards the parasite [44]. The authors reported a significantly lower number of T regulatory cells in the lungs of dogs treated with a combination of both drugs compared to dogs treated with doxycycline or ivermectin alone. It is likely that other variables are at work in eliminating the worms from the host environment. In this light, it must not be forgotten that doxycycline has numerous, non-antibiotic effects in many cells/tissues. One of the most interesting of these is the inhibition of mitochondrial biogenesis [45]. Indeed, doxycycline targets mitochondria due to the bacterial origins of this intracellular organelle. It has also been shown that many ABC transport proteins are expressed on mitochondria [46]. Therefore, doxycycline induced up- or downregulation of gene expression may indeed be due to mechanisms that are independent of its antibiotic function.

The greatest limitation to the present study is the small number of worm samples analyzed due to working exclusively with naturally-infected dogs. First, while surgical removal of adult parasites is an increasingly popular option for treatment of dogs, it is still relatively uncommon. A recent study has shown that only 3% of practitioners carry out this procedure [47]. Secondly, while experimentally-infected dogs have a predictable and substantial adult worm burden, the parasitic burden is much lower in naturally-infected dogs, and surgical removal generally yields between three to six worms per dog, according to the dog size. Lastly, it was necessary to identify dogs that had not undergone previous macrocyclic lactone and/or doxycycline treatment, from veterinary practices within a reasonable distance from the laboratory, in order to obtain live, metabolically active worms.

## Conclusions

The present study is the first, to the authors’ knowledge, to evaluate the expression of P-gP genes in adult *D. immitis*. We have described a sex-dependent differential gene expression of three *D. immitis* P-gps treated with doxycycline, ivermectin and a combination of doxycycline + ivermectin and suggest that this difference may be due to the presence of *Wolbachia*. It is likely that different exposure times and drug concentrations would give different results. Further study, therefore, is necessary to determine if modulation of cellular efflux is in part responsible for the adulticide effect of doxycycline/macrocyclic lactone combinations in heartworm-infected dogs.

## Data Availability

All data generated or analyzed during this study are included in this published article.

## Abbreviations

ABC: ATP-binding cassette
ANOVA: analysis of variance
cDNA: complementary deoxyribonucleic acid
DMSO: dimethyl sulfoxide
DOXY: doxycycline
IVM: ivermectin
HPLC: high performance liquid chromatography
ML: macrocyclic lactone
PgP: P-glycoprotein
qRT-PCR: quantitative reverse-transcription polymerase chain reaction
SEM: standard error of the mean
